# Improved accuracy of mandible geometry reconstruction at the stage of data processing and modeling

**DOI:** 10.1007/s13246-018-0664-5

**Published:** 2018-07-05

**Authors:** Grzegorz Budzik, Paweł Turek

**Affiliations:** Faculty of Mechanical Engineering & Aeronautics, Rzeszow University of Technology, Powstańców Warszawy Avenue 12, 35-959 Rzeszow, Poland

**Keywords:** Accuracy, CT image segmentation, Lanczos resampling filter, Mandible reconstruction, Slice thickness

## Abstract

The article presents a comparative study of influence of the Lanczos resampling filter on improving the accuracy of reconstruction of mandible geometries. The research was performed on eight different patients. Digital Imaging and Communications in Medicine data were obtained on the Siemens Somatom Sensation Open 40 scanner. At the stage of reconstruction, the same parameters were utilized, while only slice thickness was changed. Modeling with voxel dimensions of 0.4 mm × 0.4 mm × 1.5 mm was chosen as the gold standard over the modeling approach comprising voxel dimensions of 0.4 mm × 0.4 mm × 3.0 mm and improved using the Lanczos resampling filter. The influence of the Lanczos resampling filter on the accuracy of reconstruction of mandible geometry is very similar for the eight presented patients. The average results show a distribution with a positive skew and kurtosis. The value of skewness is 0.713 and kurtosis is 4.221 for the model without Lanczos filtering applied. When the Lanczos filtering is applied the value of skewness is 0.542 and kurtosis is 4.313. Based on 95% confidence, changes in layer thickness from 1.5 mm to 3 mm generated errors reconstructing the geometry of the mandible at the value of 0.153 mm ± 1.209 mm. In models improved using the Lanczos resampling filter, the errors generated in reconstructing the geometry of the mandible were minimized at the value of 0.160 mm ± 1.007 mm. The presented research highlights new opportunities to improve the accuracy of reconstruction geometry of the mandible at the stage of data processing.

## Introduction

Reverse Engineering (RE) is a process which allows for reconstructing the geometry of an already existing object [1]. It is applied in many areas of our lives including medicine [2, 3]. The geometry of a medical model can be reconstructed in two different ways depending on whether the measurements are conducted inside or outside of a living organism. One was is the medical method (medical path) which allows the reconstruction of the external geometry of internal anatomical structures [4, 5] in order to make surgery templates [5–7] and ready implants [8, 9]. The non-medical method (technical path) is an addition to the medical method where coordinate measuring systems are applied for verification of model accuracies used in the machining industry [10, 11], medicine [12–14] and in designing orthoses which stabilize joints [15].

Each stage of the medical method has an impact on the accuracy of a model’s geometry reconstruction. The data acquisition stage is the most important one since the appropriate selection of the system and measurement parameters influence the quality of the obtained diagnostic data [16, 17]. The stage of data processing comes next and usually begins with digital filtering to remove measurement noise appearing in the 2D images. Then, a chosen anatomical structure is separated through the application of various segmentation methods based mainly on detection of edges and identification of image areas with some common features (e.g. brightness, gray levels etc.) [18–20]. Volume data may be visualized in the form of a 3D model by using indirect and direct methods. In the case of Computer Aided Design (CAD) modeling, indirect surface methods are usually used. An isosurface is constructed from a particular scalar field and then a surface is made of voxels with a set value by using conventional methods of graphics rendering on the basis of polygon grids [12, 21]. These reconstructed geometries require additional editing such as inversion of surface normals or removal of gaps between surfaces [22, 23]. A finished model may be manufactured by using additive technologies [12, 14, 24] or covered the polygonal grid with elementary NURBS surfaces [25]. The obtained model may be exported in various CAD data exchange formats such as *.iges, and *.step. Also, it may be used as a model for creating a processing program in Computerized Numerical Control (CNC) machines [13].

Currently, scientists are working on methods to improve the quality of medical manufacturing models using Rapid Prototyping (RP) [12, 14, 22, 24] and CNC techniques [13]. A particularly difficult task is to determine and improve the accuracy of digital models at the stage of acquisition and data processing. Slice thickness plays an important role in volume averaging, thereby affecting spatial resolution in the image. Changing the slice thickness can create an artifact–partial-volume effect. This is a process by which different tissue attenuation values are averaged to produce one less accurate pixel reading. Partial volume effects are omnipresent in medical image data and must, therefore be taken into account for segmentation, classification, quantitative image analysis [3, 26]. This artifact directly can affect volume, geometry and linear accuracy of digital and physical 3D models. CT scanners installed in clinics usually produce data with an anisotropic structure of voxels. This irregular type of data produces errors in the process of interpretation of pathology on 2D images and in full 3D visualization. The low visual quality that results is due to the discontinuous interpolation results between neighboring voxels, resulting in a very blocky appearance of the reconstructed surfaces (staircase artifact) [3].This structure generates geometry errors, which influence the final accuracy of manufactured biomodels. Currently, scientists are working on precisely determining the deviation and distribution associated with a change in slice thickness for reconstruction of geometry [21, 27] but none actually presents methods to improve the spatial resolution of this data at the stage of data processing. The article presents a method which may improve the design and manufacture of mandible models and extend the methodology of analytical accuracy at the stage of data acquisition.

## Method

The research was performed on eight different patients. Digital Imaging and Communications in Medicine (DICOM) data were obtained on the Siemens Somatom Sensation Open 40 scanner installed in the Regional Clinical Hospital No. 1 for all patients at the Frederic Chopin in Rzeszow, with the scanning protocol ‘‘Head Routine’’. At the stage of reconstruction, the same parameters were used, while only slice thickness was changed (Table 1). A stack of images were loaded into the Amira software. The key to converting anatomical data from images to 3D models is a process called segmentation. To extract the mandible model from DICOM data, a region-growing algorithm was used. Thresholds were set above 200HU to select only craniofacial tissue. After a 3D image was segmented, a marching cubes algorithm was used for computing isosurfaces. This algorithm proceeds through the scalar field, taking eight neighbor locations at a time (thus forming an imaginary cube), then determining the polygon(s) needed to represent the part of the isosurface that passes through this cube. The individual polygons are then fused into the desired surface. This is done by creating an index to a recalculated array of 256 possible polygon configurations within the cube, by treating each of the eight scalar values as a bit in an 8-bit integer. If the scalar’s value is higher than the iso-value (i.e., it is inside the surface), then the appropriate bit is set to one, while if it is lower (outside), it is set to zero. The final value, after all, eight scalars are checked, is the actual index to the polygon indices array. Finally, each vertex of the generated polygons is placed on the appropriate position along the cube’s edge by linearly interpolating the two scalar values that are connected by that edge. The reconstructed surface was free from cracks and holes. The final surface was saved in stereolithography (STL) file, which represents the 3D model.

**Table 1:** Scanning protocol

Somatom Sensation 40 (spiral mode)		
Name of parameters	Value of parameters (first measurements)	Value of parameters (second measurements)
kV	120	120
mAs	380	380
Rotation time	1 s	1 s
Acquisition	24 × 1.2 mm	24 × 1.2 mm
Slice collimation	1.2 mm	1.2 mm
Kernel	H60s	H60s
Matrix size	512 × 512	512 × 512
Pixel size	0.4 mm × 0.4 mm	0.4 mm × 0.4 mm
Slice thickness	1.5 mm	3 mm

Improved spatial resolution of DICOM data was achieved in this study with the use of a Lanczos resampling filter. It is an interpolation function that is used extensively in the arena of digital signal processing. It is basically a Fourier kernel. Its essentiality is for smoothly interpolating the value of a digital signal between its samples. Each of the given signal’s samples is effectively mapped to give a translated and scaled copy of the Lanczos kernel. A Lanczos kernel is nothing but a sinc function apodized by the central hump of a dilated sinc function [1]. The sum of these shifted and scaled kernels are then evaluated at the requisite points. Lanczos resampling is also referred to as Lanczos filter. Lanczos resampling finds application for incrementing the sampling rate of a digital signal. It finds application in digital image processing for performing multidimensional interpolation, and it provides excellent results amongst several filters that exist in the literature [28].

**Fig. 1:**
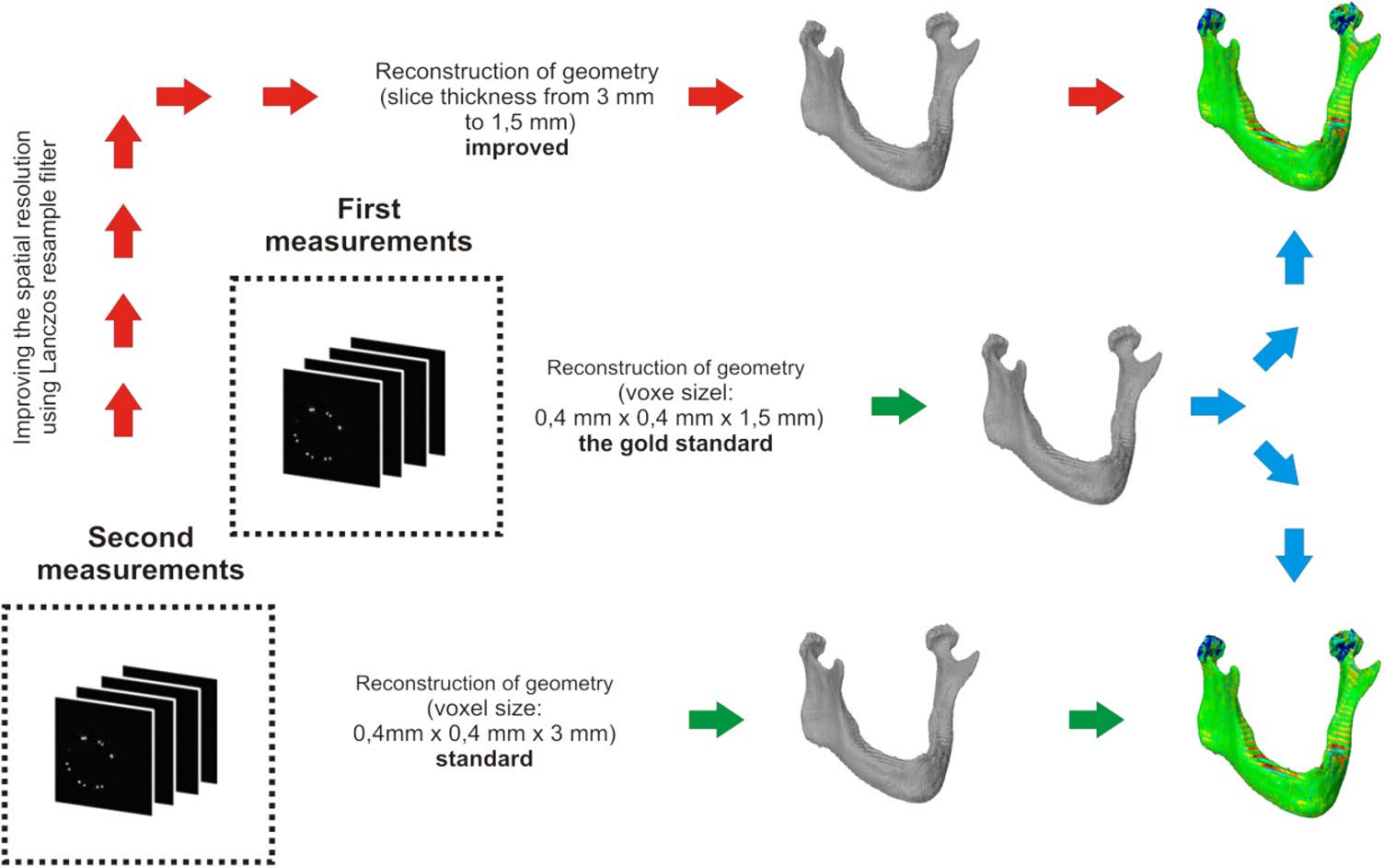
The comparison process

The improved spatial resolution was done to reformat a voxel’s structure from 0.4 mm × 0.4 mm × 3 mm to 0.4 mm × 0.4 mm × 1.5 mm. Then the digitally processed data were subjected to the process of data segmentation and geometry reconstruction under the same methods and parameters as the non-processed models obtained from the first and the second measurements. The model with voxel dimensions of 0.4 mm × 0.4 mm × 1.5 mm was chosen as the gold standard over the model reconstructed with voxel dimensions of 0.4 mm × 0.4 mm × 3 mm and improved using the Lanczos resampling filter (Fig. 1). The model with voxel dimensions of 0.4 mm × 0.4 mm × 1.5 mm (first measurements) was chosen as the gold standard because a single voxel had the smallest volume relative to the reconstructed model with voxel dimensions of 0.4 mm × 0.4 mm × 3 mm (second measurements). In addition, an artifact–partial-volume effect and artifact-associated errors in the interpolation data were minimized in this model.

Comparison process was carried out in Focus Inspection software using a best-fit algorithm, which is a most appropriate algorithm for analyzing deviations in models with complex shapes [29–31]. A best-fit alignment is an iterative process using the condition of minimizing the square of the distance between the nominal and measured data to converge on a solution. Adjustment of point clouds using the best-fit in this article was carried out to an accuracy of 0.001 mm. This minimal improvement parameter represents the criteria used to determine when the best-fit alignment is achieved. If the movement required during any one iteration is greater than this value, then further iterations will continue until the movement is less than the specified value. Evaluation of the quality of reconstruction geometry was carried out using conventional and positional measurements describing the structure of the community. Mean deviation, standard deviation (SD), skewness and kurtosis were taken into consideration. In the case of a medium or large asymmetry occurring, the median and mode were also determined. The purpose of this was to consider the relationship between the mode and median and the resulting mean deviation. The average values were used as the final results for the 8 patients.

## Results

**Fig. 2:**
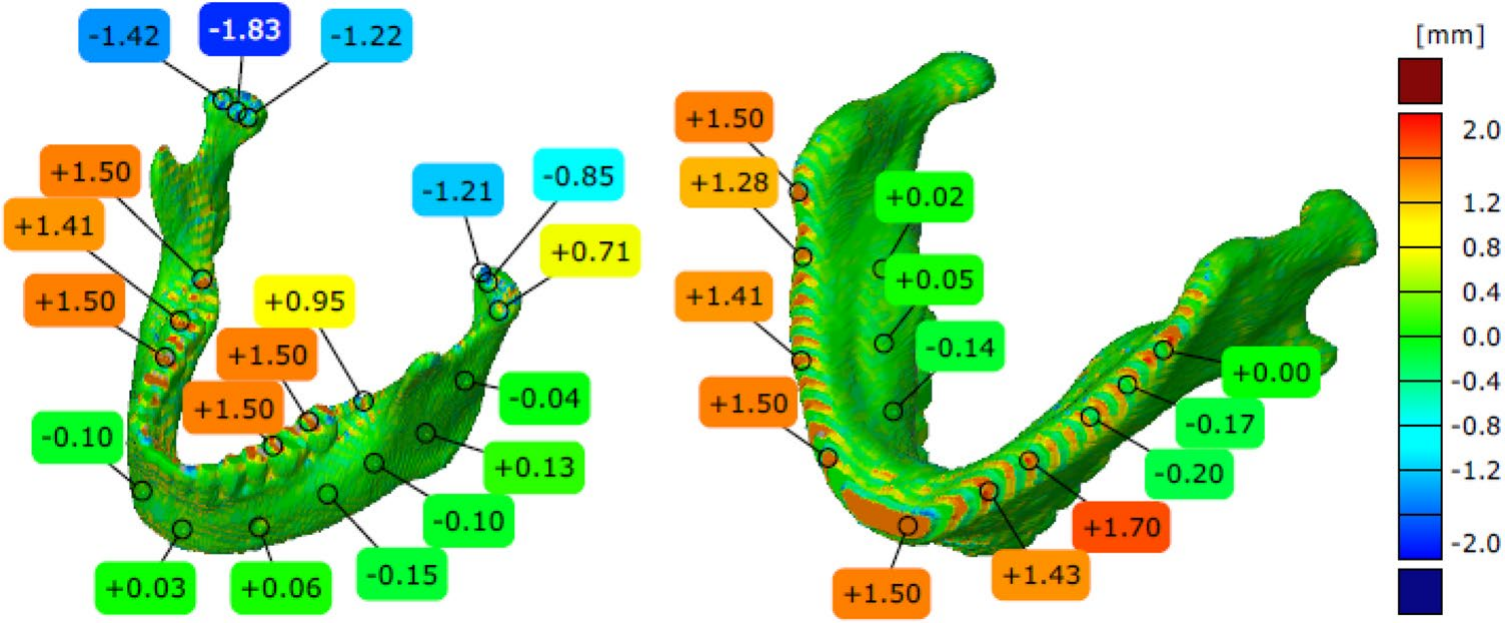
Maximum positive and negative deviations for the second patient (not improved)

**Fig. 3:**
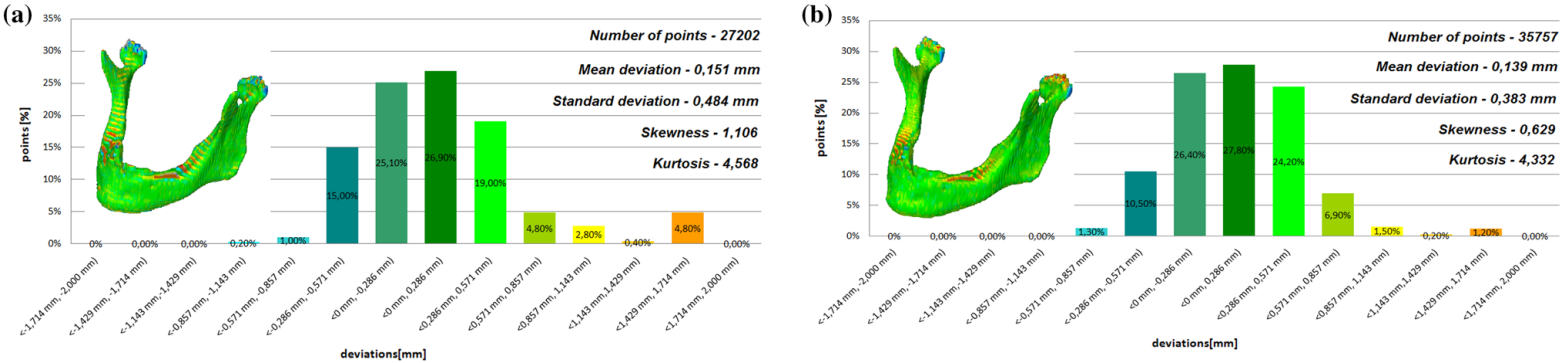
Histogram of the first patient **a** not improved, **b** improved using the Lanczos resampling filter

**Fig. 4:**
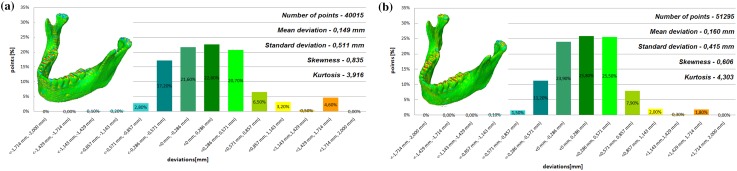
Histogram of the second patient **a** not improved, **b** improved using the Lanczos resampling filter

**Fig. 5:**
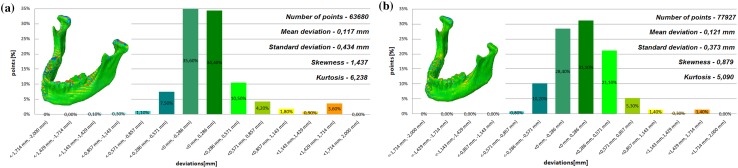
Histogram of the third patient **a** not improved, **b** improved using the Lanczos resampling filter

**Fig. 6:**
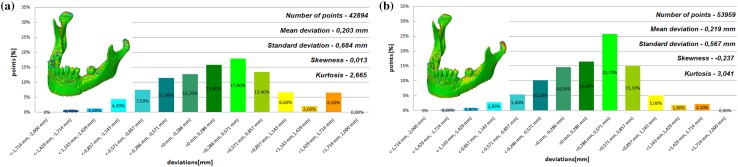
Histogram of the fourth patient **a** not improved, **b** improved using the Lanczos resampling filter

**Fig. 7:**
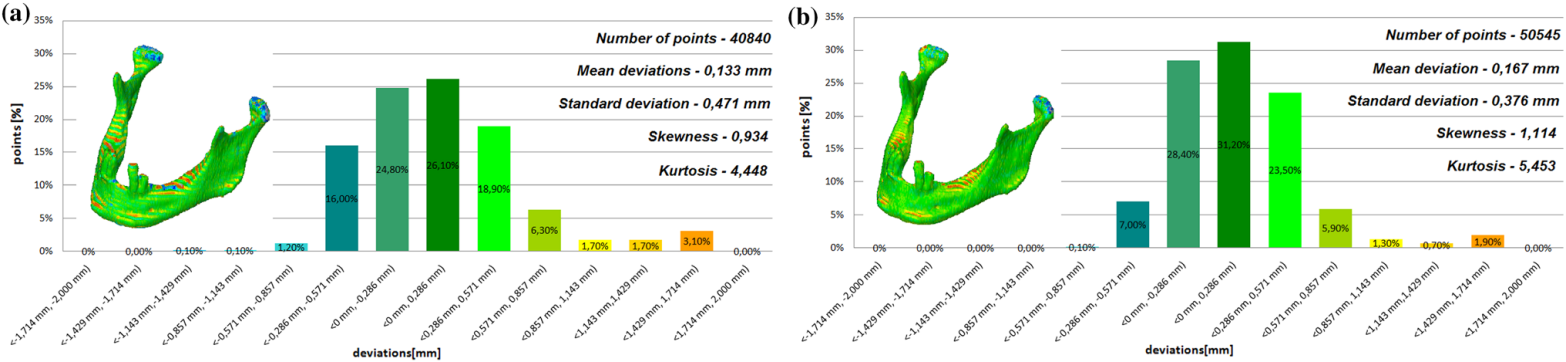
Histogram of the fifth patient **a** not improved, **b** improved using the Lanczos resampling filter

**Fig. 8:**
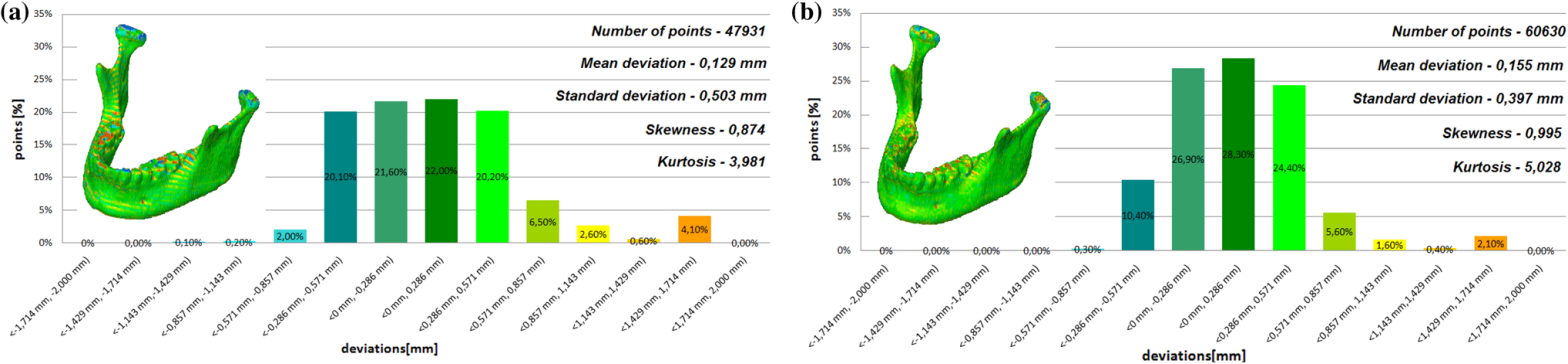
Histogram of the sixth patient **a** not improved, **b** improved using the Lanczos resampling filter

**Fig. 9:**
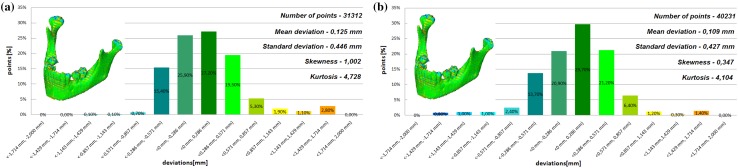
Histogram of the seventh patient **a** not improved, **b** improved using the Lanczos resampling filter

**Fig. 10:**
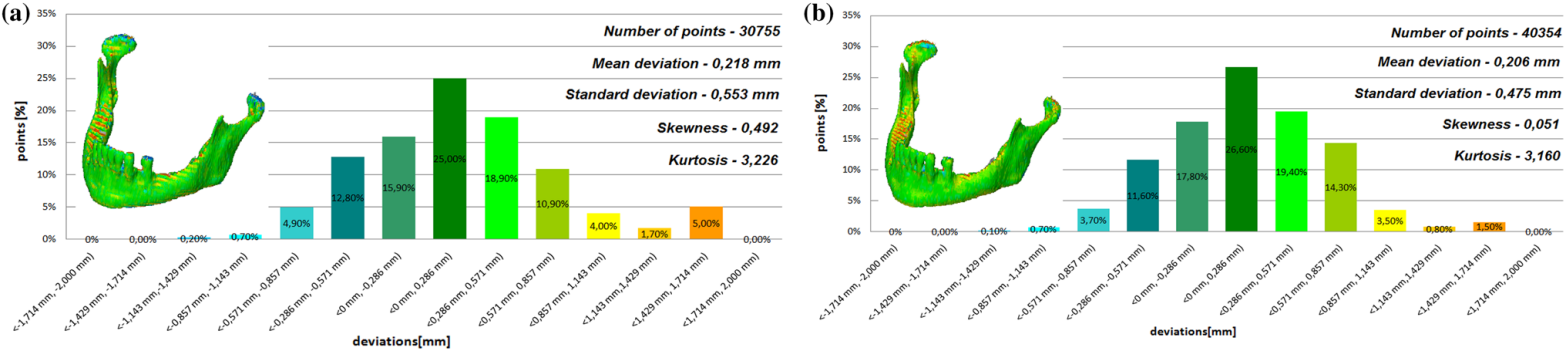
Histogram of the eighth patient **a** not improved, **b** improved using the Lanczos resampling filter

The models of the eight mandibles with a voxel size of 0.4 mm × 0.4 mm × 3 mm and improved using Lanczos resample filter were compared with the same mandible based on modeling with a voxel size of 0.4 mm × 0.4 mm × 1.5 mm to examine the influence the Lanczos resampling filter on the accuracy of reconstruction. In the most analyzed mandible, the maximum positive and negative deviations occurred in the same region. Figure 2 presents the maximum positive and negative deviations for the second patient. The statistical parameters and distributions of the reconstructed models from DICOM data are presented in Figs. 3, 4, 5, 6, 7, 8, 9, 10. In the process of assessing the normality of the data by the Shapiro-Wilks test (Figs. 3, 4, 5, 6, 7, 8, 9, 10), the value of the statistics test was W = 0.836 (p = 0.05 and n = 8) in models not improved and in models improved was W = 0.818 (p = 0.05 and n = 8). This value is higher than the critical value, and as a result, we did not reject the hypothesis of the normal distribution. Accordingly, all obtained distributions were treated as normal.

The poor quality of this region of geometry is due to the lack of continuity of data interpolation, which in turn generates the block structure of the model (staircase artifact). This artifact mostly generates positive deviations in the analyzed mandible. There are also spots where negative deviations can be observed. In most cases, their values are within the range of 30–40% in relation to the whole populace of the studied points. The influence of the Lanczos resampling filter on the accuracy of reconstruction of mandible geometry is very similar for the 8 presented patients. In the case of Lanczos filtering compared to the whole data population, there was a reduction in the amount of positive deviations within the tolerance range of < 0.857 mm; 1.714 mm > by about 5% and negative deviations < − 0.857 mm; − 0.286 mm > by about 2–12%, for the eighth and sixth model, accordingly. In the case of positive deviations, this result is mainly connected with reduction of the staircase artifact resulting from improvement of data interpolation. When it comes to the negative deviations (which are located mainly within the temporomandibular joint), their reduction results from, among others, increased accuracy of segmentation in this area of the mandible. One may also see an improvement in obtained values of standard deviation. Despite the increase in a number of points reflecting the processed models’ geometry, the standard deviation was reduced by over 0.1 mm. When the Lanczos filtering is applied, eight distributions show positive skew and kurtosis. The value of skewness is in the range from 0.013 to 1.437, and kurtosis from 2.665 to 6.238. In the case of skew and kurtosis, this demonstrates small, medium to high asymmetry and a distribution is more peaked than a Gaussian distribution (Leptokurtic distribution). Patient 4 (see Fig. 6a) was the only one to show platykurtic distribution. When the Lanczos filtering is applied, seven distributions show positive skew and kurtosis only distribution presented in Fig. 7b show negative skew. The value of skewness is in the range from − 0.237 to 1.114, and kurtosis from 3.04 to 5.453. In the case of skew and kurtosis, this demonstrates medium to high asymmetry and a distribution is more peaked than a Gaussian distribution. In most cases, use of Lanczos filtering reduced asymmetry intensity (in some cases from strong to medium) and increased the value of kurtosis. For this reason, we calculated also the mode and median values of data (Table 2). The relationship between the value of the mode and median and the resulting mean value further confirms the existence of positive skew.

**Table 2:** Median and mode value

Model number	Not improved		Improved using Lanczos filtering	
	Median (mm)	Mode (mm)	Median (mm)	Mode (mm)
First	0.091	0.051	0.122	0.079
Second	0.102	0.099	0.146	0.124
Third	0.047	0.016	0.097	0.06
Fourth	0.210	0.211	0.309	0.324
Fifth	0.083	0.042	0.132	0.075
Sixth	0.08	0.01	0.125	0.072
Seventh	0.08	0.037	0.091	0.08
Eighth	0.179	0.169	0.2	0.197
**Average**	**0.105**	**0.079**	**0.153**	**0.126**

**Fig. 11:**
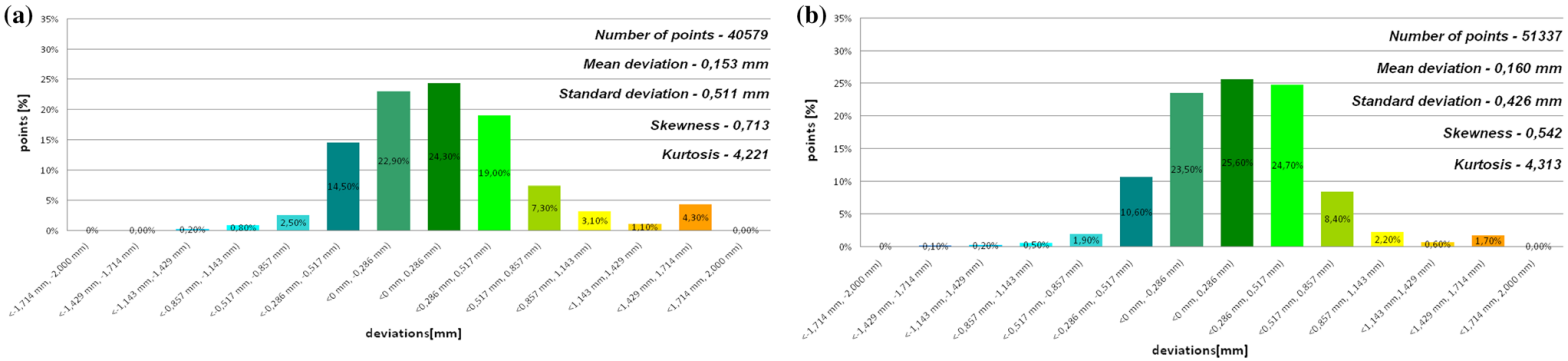
Histogram representing the average of the 8th patient, **a** not improved, **b** improved using the Lanczos resampling filter

The average values and distributions are presented in Fig. 11a, b. In Fig. 11a, b, the distributions show positive skew and kurtosis. The value of skewness is 0.713 (high asymmetry) and kurtosis is 4.221 (a distribution more peaked than a Gaussian distribution) for the model without Lanczos filtering. When the Lanczos filtering has applied the value of skewness is 0.542 (medium asymmetry) and kurtosis is 4.313. We also determined the value of the median and mode to consider the relationship between them and the resulting mean value. All presented values confirmed that distribution is characterized by positive skew in improved and not improved the model.

The process of assessing the normality and noted values of mean and SD (Fig. 11 a and b), allowed calculation of uncertainty at the level p = 0.05 (k = 2.365 for n = 8). The value of uncertainty was 1.209 mm in models not improved and 1.007 mm when the Lanczos filtering is applied. This calculated value for the change in layer thickness from 1.5 to 3 mm on the Somatom Sensation Open 40 scanner, generated errors reconstructing the geometry of the mandible at the value of 0.153 mm ± 1.209 mm. In models improved using the Lanczos resampling filter, the errors generated in reconstructing the geometry of the mandible were minimized at the value of 0.160 mm ± 1.007 mm.

## Discussion

The accurate reconstruction process of geometry is depend on type of CT scanner [16, 17], method of segmentation [19, 20], software algorithm [12, 21] and manufacturing technology [12, 13]. Currently, scientists are working on precisely determining the deviation and distribution associated with a change in slice thickness for reconstruction of geometry [21, 27] but none actually presents methods to improve the spatial resolution of this data at the stage of data processing. The spatial resolution defines the ability to resolve, as separate forms, small objects that are very close together (like in the area of the temporomandibular joint). Most datasets in medical imaging are anisotropic, and in many cases, the slice thickness is several times larger than the pixel distance [3, 16]. Changing the slice thickness can create an artifact–partial-volume effect. This is a process by which different tissue attenuation values are averaged to produce one less accurate pixel reading. Partial volume effects are omnipresent in medical image data and must, therefore, be taken into account for segmentation, classification, quantitative image analysis and final reconstruction 3D geometry.

**Fig. 12:**
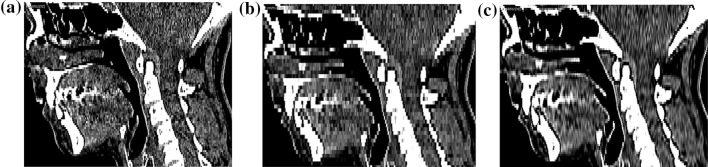
View on the 2D image of the second patient **a** voxel size: 0.4 mm × 0.4 mm × 1.5 mm, **b** voxel size: 0.4 mm × 0.4 mm × 3 mm, **c** improved using the Lanczos resampling filter

Figure 12a, b present 2D images show an impact of the layer’s thickness on the quality of obtained tomographic data. In the case of the second measurement, in which the volumetric data were characterized by voxel dimensions 0.4 mm × 0.4 mm × 3 mm, it can be seen that there is a major impact of the staircase artifact which significantly hindered the process of the mandible’s segmentation. The segmentation problem occurred in particular at the area of the teeth crowns (separation of the mandible from the maxilla) as well as the area of the temporomandibular joint (separation of the mandible from the cranium). Eventually, the mandible’s geometry was reconstructed from the data obtained in this way but this process required a lot of work and time. As a result of such problems, the data with a voxel structure 0.4 mm × 0.4 mm × 3 mm were subjected to the improvement of data spatial resolution. In this case, data resampling with the Lanczos filtering was used. The influence of the staircase artifact was significantly minimized (Fig. 12c) using the filtered data in comparison to the data with a voxel’s structure 0.4 mm × 0.4 mm × 3 mm (Fig. 12b). The process of mandible segmentation, which has been conducted on such digitally processed data, also required less effort and time on the filtered data.

The influence of an anisotropic structure of voxel on the accuracy of mandible geometry is very similar for the eight presented patients. This artifact mostly generates positive deviations in the area of the mandibular body (see Fig. 2). There are also spots where negative deviations can be observed (in the area of the condyle). When using the Lanczos resampling filter, one may observe an improvement in the accuracy of the reconstruction in the area of the mandibular body (positive deviations were minimized) and segmentation in the area of the condyle (negative deviations were minimized). This is also confirmed by the statistical parameters. The standard deviation was reduced by over 0.1 mm. In the case of the mean deviation, the value a slight increase. This is related to the fact that using the Lanczos resampling filter, mainly minimizes deviations with a negative value than a positive one (segmentation process was improved in the area of the condyle). Results presented in Figs. 3, 4, 5, 6, 7, 8, 9 and 10 prove that on the stage of data editing, it is possible to increase the accuracy of segmentation and reconstruction process of the geometry of the mandible.

## Conclusion

This research showed that the spatial resolution, which is determined by the layer thickness, has a major impact on the accuracy of reconstruction of 3D models. The resolution of DICOM data must be chosen very carefully to minimize the problem of partial volume effects and staircase artifacts. The influence of the Lanczos resampling filter on the accuracy of reconstruction of mandible geometry is very similar for the eight presented patients. When using the Lanczos resampling filter, one may observe an improvement in the accuracy of the reconstruction of the mandible’s geometry. Also, the time of segmentation was shortened (in particular of the temporomandibular joint from the cranium). The presented results prove that on this stage of data editing, it is possible to increase the accuracy of segmentation and reconstruction of the geometry of the mandible using the Lanczos resampling filter. The resampling process can be used to improve DICOM data with a different value of slice thickness. It is a starting point in a focus on creating software that controls deviations during reconstruction and supports designers in modeling 3D geometry obtained from the most popular Cone and Multi-Detector Computer Tomography scanners.
